# Pulse-Based Nutrition Education Intervention Among High School Students to Enhance Knowledge, Attitudes, and Practices: Pilot for a Formative Survey Study

**DOI:** 10.2196/45908

**Published:** 2023-05-31

**Authors:** Getenesh Berhanu Teshome, Hiwot Abebe Haileslassie, Phyllis Shand, Yun Lin, Jessica R L Lieffers, Carol Henry

**Affiliations:** 1 College of Pharmacy and Nutrition University of Saskatchewan Saskatoon, SK Canada; 2 Applied Human Sciences University of Prince Edward Island Charlottetown, PE Canada

**Keywords:** adolescents, food literacy, high school, macronutrients, micronutrients, pulses, dietary pattern, diet, eating habits, nutrition, students, school-based interventions

## Abstract

**Background:**

Promoting pulse consumption in schools could improve students’ healthy food choices. Pulses, described as legumes, are rich in protein and micronutrients and are an important food choice for health and well-being. However, most Canadians consume very little pulse-based food.

**Objective:**

This pilot study sought to investigate outcomes of a teacher-led, school-based food literacy intervention focused on the Pulses Make Perfect Sense (PMPS) program in 2 high schools in Saskatoon, Saskatchewan.

**Methods:**

Both high schools were selected using a convenience sampling technique and have similar sociodemographic characteristics. The mean age of students was 16 years. The intervention comprised 7 key themes focused on pulses, which included defining pulses; health and nutritional benefits of pulses; incorporating pulses into meals; the role of pulses in reducing environmental stressors, food insecurity, and malnutrition; product development; taste testing and sensory analysis; and pulses around the world. A self-administered questionnaire was used to assess knowledge, attitudes, practices, and barriers regarding pulse consumption in students at baseline and study end. Teachers were interviewed at the end of the intervention. Descriptive statistics and the nonparametric Mann-Whitney *U* test were used for analysis.

**Results:**

In total, 41 and 32 students participated in the baseline and study-end assessments, respectively. At baseline, the median knowledge score was 9, attitude score was 6, and barrier score was 0. At study end, the median knowledge score was 10, attitude score was 7, and barrier score was 1. A lower score for barriers indicated fewer barriers to pulse consumption. There was a significant difference between baseline and study-end scores in knowledge (*P*<.05). Barriers to pulse consumption included parents not cooking or consuming pulses at home, participants not liking the taste of pulses, and participants often preferring other food choices over pulses. The teachers indicated that the pulse food-literacy teaching resources were informative, locally available, and easy to use.

**Conclusions:**

Despite the improvements in knowledge, attitude, and practice, pulse consumption did not change significantly at the end of the intervention. Future studies with larger samples are needed to determine the impact of PMPS on knowledge, attitude, and practice of high school students.

## Introduction

Achieving a healthy dietary pattern requires properly planning, choosing, and preparing a variety of foods. However, reports have shown a gap in food knowledge and the skills required to achieve healthy dietary patterns in the current complex food system [1]. A systematic review by Vaitkeviciute et al [2] suggested that food literacy could improve food-related skills and knowledge, thereby contributing to long-lasting healthy dietary behaviors among adolescents [3].

Food literacy is defined as “the scaffolding that empowers individuals, households, communities or nations to protect diet quality through change and strengthen dietary resilience over time. It is composed of a collection of inter-related knowledge, skills, and behaviors required to plan, manage, select, prepare and eat food to meet needs and determine intake” [4]. There is growing evidence that cooking skills, which are a component of food literacy, are linked with healthier dietary choices, such as increased intake of fruits, vegetables, and whole grains [5]. Adolescents who had a higher level of food literacy were found to have a healthier dietary intake [2].

Increasingly, attention is being paid to consuming plant-based food products, including pulses, which comprise dry beans, lentils, chickpeas, and peas. Recognizing their health benefits, the most recent version of Canada’s Food Guide, released in 2019, emphasizes the consumption of pulses [6]. Pulses are rich in protein, micronutrients, and fiber and low in fat. Pulses also have a low glycemic index. Although Canada produces and exports pulse crops, most Canadians consume very little pulse-based food. A study that analyzed pulse and soy consumption using Canadian Community Health Survey (CCHS) 2.2 data for Manitoba residents found that only 4.4% of adolescents aged 14 to 18 years consumed pulses or soy on any given day, based on 24-hour recall [7]; this study suggests that there is a much lower intake of pulses among adolescents relative to pulse intake among adults at a national level (13%) [8].

Adolescence is a period of rapid growth with increased nutrient requirements that makes this group nutritionally vulnerable. It is also a transition period to adulthood, when most habits are formed [3,9,10]. Healthy eating habits that are created in childhood and in adolescence are critical to establishing healthy eating behaviors that persist into adulthood [11]. Common eating behaviors during adolescence include skipping meals, eating food away from home, high consumption of sweets, and consumption of nutrient-poor snacks that usually fail to meet nutrition recommendations [2,10]. A study done in Canada found that 50% of grade 9 to 12 students skipped breakfast at least once per week and ate fast foods twice per week or more [12]. These behaviors are associated with poor diet quality and can affect nutrient intake in adolescents [12,13].

To date, there are limited studies that have targeted nutrition education and cooking skills in the high school setting. School-based interventions that combine hands-on cooking and nutrition education can have a long-term impact on adolescent progress toward healthy dietary choices [14,15]. Increased preference for fruits and vegetables and cooking self-efficacy have been reported in programs that integrate cooking and nutrition education [16]. This pilot study sought to investigate the effect of nutrition education intervention on high school student knowledge, attitudes, and practices related to pulse consumption in Saskatoon, Saskatchewan.

## Methods

### Theoretical Framework

The food literacy framework used in this study consisted of several interconnected attributes, including food knowledge, nutrition knowledge, food and nutrition language, food skills, nutrition literacy, food and nutrition self-efficacy, cooking self-efficacy, food attitude, food and other systems, social determinants of health, and dietary behavior, under the categories of food and nutrition knowledge, food skills, self-efficacy and confidence, food decisions, and ecological (ie, external) factors, such as income security and the food system [17]. Appropriate knowledge, skills, and the ability to apply them are important to achieve food literacy at individual and community levels. The food literacy framework helps adolescents to make meaningful dietary changes by interconnecting food, health, and the environment. This study focused on food literacy to improve adolescent knowledge, dietary behavior, and attitudes toward healthful food choices, specifically pulses.

### Intervention Design and Setting

The Pulses Make Perfect Sense (PMPS) nutrition education intervention was guided by concepts from the food literacy framework [18]. The PMPS intervention targets various categories of food literacy, such as food and nutrition knowledge, food skills, self-efficacy and confidence, food decisions, and ecological (ie, external) factors (Table 1). The intervention in this pilot study was offered over a 7-week period to grade 10, 11, and 12 students taking food studies and home economics–commercial cooking classes at 2 purposively selected high schools in Saskatoon, Saskatchewan.

Both high schools (school A and school B) were selected using a convenience sampling technique [18] and have similar sociodemographic characteristics. A teacher from each school who taught the courses listed above agreed to participate in the design and implementation of the PMPS intervention. The 2 teachers also led the delivery of the pilot intervention in their classrooms. The research team contacted both of the teachers for the first time in 2018, and the intervention was delivered from October to November 2019. It is worth noting that incorporating educational activities surrounding pulses was not new to these teachers. However, incorporating pulse-related activities using a food literacy framework was new to them.

Many of the materials used in the development of the PMPS nutrition education resources were adapted with permission from elsewhere, including International Year of Pulses education packs developed by Agriculture in the Classroom Canada and Pulse Canada [19]. The educational sessions were designed to be delivered in 45 to 60 minute blocks. The delivery of the intervention was primarily teacher-led, but graduate students, a postdoctoral fellow, and an undergraduate student in education also helped to deliver the intervention. Community partners provided pulses in bulk to the schools for use in the various recipes prepared during class sessions. High school students were also encouraged to take packages of pulses home to their families. Details of the PMPS lesson themes and classroom activities can be found in Table 1.

During the intervention, teachers provided updates to researchers each week. Each teacher took a slightly different approach in providing the intervention to their students. The differences are outlined below and in Table 1.

**Table 1:** Key themes, content, activities, and corresponding food literacy categories of the Pulses Make Perfect Sense (PMPS) nutrition education intervention.

Key lesson themes	Content [20,21]	Activities	Food literacy categories [17]
Defining pulses	Pulses are edible seeds of plants in the legume family Pulses include dry peas, chickpeas, dry beans, and lentils Visual guide showing the different types of pulses Other uses of pulses: animal feed and soil improvement Pulses in Canada	Classroom discussion Both schools discussed health claims regarding protein foods	Food and nutrition knowledge
Health and nutritional benefits of pulses	Health benefits of consuming pulses Nutritional benefits of consuming pulses: protein, micronutrients, fiber Pulses and Canada’s Food Guide	Lentil smoothies School A: Taste-tested lentil smoothies School B: Prepared and taste-tested lentil smoothies	Food and nutrition knowledge Food skills
Incorporating pulses into meals	Preparing dry and canned pulses Incorporating pulses into various dishes Cooking pulses in a pressure cooker or slow cooker Safety during cooking Buying and storing pulses	Taco in a bag School A: Taste-tested taco in a bag School B: Prepared and taste-tested taco in a bag	Food skills Cooking self-efficacy and confidence Food and nutrition knowledge
The role of pulses in reducing environmental stressors	How do pulse plants grow? Soil health Symbiosis Nitrogen fixation Intercropping Crop rotation Pot planting	Planting pulses into pots Both schools A and B planted pulses into pots School B: Cooked lentil muffins	Ecological (ie, external) factors
The role of pulses in reducing food insecurity and malnutrition	Food security Cost	Cook budget-friendly pulse-based dishes School A: Cooked soup mix School B: Cooked pulse sauce and pasta and soup mix	Food skills Cooking self-efficacy and confidence Ecologic (external) factors Food and nutrition knowledge
Product development, taste testing, and sensory analysis	Pulse product development and tasting	Taste-tested commercially prepared product made from meat-pulse hybrid (meat-pulse bar) Both schools taste-tasted meat-pulse bar	Ecological (external) factors Food skills Food and nutrition knowledge
Pulses around the world	Ways in which pulses are used in appetizers, snacks, and side dishes and main dishes in various countries Pulses as vegetarian and vegan offerings Incorporating pulses into Saskatchewan-based dishes	Lentil bits School A: Taste-tested lentil bits School B: Cooked and taste-tested lentil bits	Food and nutrition knowledge Food skills Food decisions

### School A

The pilot intervention in school A was delivered as part of the food studies class. The students received both the theory and cooking lab exercises once a week during the same class period for 7 weeks. One postdoctoral fellow and one nutrition graduate student from the University of Saskatchewan (USask) assisted in facilitating the activities (Table 1). Given the limited food preparation space, some food items were prepared in the undergraduate food lab at USask and brought to the school for serving.

### School B

In school B, students taking the home economics–commercial cooking class received both the theory and cooking exercises over 2 class periods each week. The first class involved the delivery of activities related to theory and the second class was devoted to practical applications and lab cooking exercises (Table 1). At school B, most of the content was taught by the teacher, except lessons 4 (the role of pulses in reducing environmental stressors) and 6 (pulses around the world), which were taught by a postdoctoral fellow and a nutrition graduate student from USask. The teacher at school B was assisted by a practicum student from the College of Education at USask with interest and experience in teaching food skills.

### Data Collection

#### Quantitative Data

Quantitative data were collected from the high school students using paper-based self-administered questionnaires 1 week prior to the beginning of the intervention (baseline) and 1 week following the intervention (study end). The questionnaires were completed in the classroom. The questionnaires were adapted from a previous questionnaire pretested in elementary schools in Saskatchewan [20,22,23] and further revised by Malik [24]. The goal of the self-administered questionnaires was to assess student knowledge, attitude, and practices and barriers to pulse consumption. In addition, students reported the frequency of consumption of pulse-based foods in the past month and the type of pulse they most often consumed. Questions to capture demographic data, such as age and sex, were also included in the questionnaire.

#### Qualitative Data

Teachers and the education practicum student involved in the delivery of the intervention were interviewed using a semistructured interview guide (Multimedia Appendix 1) after the intervention by a postdoctoral fellow. These interviews helped to assess educators’ experiences in delivering the PMPS intervention. The interviews also captured information on how to enhance student engagement, improve resources, and better accommodate the lessons during class time.

### Data Analysis

Quantitative data were analyzed using SPSS (version 26.0; IBM Corp). Analysis included descriptive statistics and the Mann-Whitney *U* test for the knowledge, attitude, and practices data. All data are presented as percentages or medians. For the knowledge questions, a score of 1 was assigned when the question was answered by the students correctly, and a score of 0 was assigned when the question was answered incorrectly. The total knowledge score is the sum of the scores. A higher score (maximum 11) indicates a greater knowledge about pulses. For questions regarding attitudes, students were asked about their perceptions about consuming pulses. All attitude questions were assigned a score of 1 for “agree” and “strongly agree,” and 0 for “disagree,” “strongly disagree,” and “not sure.” The total attitude score is a sum of the scores. A higher score (maximum 9) indicates a greater level of positivity toward pulse consumption. Participants were also asked questions about a series of possible barriers to pulse consumption. All of the barrier questions were scored the same way as the attitude questions, and a total barrier score was also calculated, with the highest possible score being 5.

Pulse consumption practices were measured using a short food frequency questionnaire. This food frequency questionnaire captured information on consumption of pulse-based foods in the past month using closed questions. A total of 10 pulse-based foods were included in the food frequency questionnaire.

Interviews were audio-recorded and transcribed verbatim by the postdoctoral fellow. The transcribed interviews were sent to the interviewees for comment and approval [25]. An inductive thematic analysis of data was used to analyze the interviews [26]. Coding was performed manually, and the coded data were categorized into different themes and subthemes. The generated themes were reviewed and refined by the study’s principal investigator for consensus validation.

### Ethical Approval

Ethical approval was obtained from the Behavioral Research Ethics Board (BEH-241) at USask. A letter of explanation that described the purpose of the intervention was provided to the principals of schools A and B. Students younger than 18 years were required to obtain parental consent to participate in the study. In addition, assent was obtained from each student.

## Results

### Characteristics of Participants

In total, 48 students from schools A and B participated in the PMPS intervention. In total, 41 students (school A: n=29; school B: n=12) at baseline and 32 students (school A: n=20; school B: n=12) at study end provided consent to participate in the study. The age range of the students at baseline was 15 to 19 years, with a mean age of 16 years. In total, 4 (10%), 24 (58%), and 13 (32%) students at baseline were in grades 10, 11, and 12, respectively. Most participants were male (27/41, 66%). Table 2 summarizes the demographic characteristics of the student participants at baseline.

**Table 2:** Demographic characteristics of student participants at baseline (n=41).

Variables			Participants, n (%)
**Age range (years)**			
	15-16	26 (63)	
	17-19	15 (37)	
**Sex**			
	Male	27 (66)	
	Female	14 (34)	
**Grade**			
	10	4 (10)	
	11	24 (58)	
	12	13 (32)	

### Knowledge About Types of Pulses and Their Benefits

Information about participant knowledge is presented in Table 3. A higher score (maximum 11) indicates a greater knowledge about pulses. The median knowledge scores at baseline and study end for all participants were 9 and 10, respectively (*P*=.006). At baseline (n=41), 98% (n=40), 93% (n=38), and 37% (n=15) of participants knew that pulses are a good source of protein, fiber, and iron, respectively. At study end (n=32), 97% (n=31), 100% (n=32) and 69% (n=22) knew that pulses are a good source of protein, fiber, and iron, respectively. At baseline (n=41), 93% (n=38), 83% (n=34), 66% (n=27), and 54% (n=22) could identify pictures of beans, peas, chickpeas, and lentils, respectively. At study end (n=32), 100% (n=32), 75% (n=24), 88% (n=28), and 81% (n=26) could identify pictures of beans, peas, chickpeas, and lentils, respectively.

**Table 3:** Knowledge of students regarding pulses and their benefits at baseline and study end.

		Baseline (n=41), n (%)	Study end (n=32), n (%)	*P* value^a^	
**Pulses are a good source of protein**					.96
	True^b^	40 (98)	31 (97)		
	False	1 (2)	1 (3)		
**Pulses are a good source of fiber**					.09
	True^b^	38 (93)	32 (100)		
	False	3 (7)	0 (0)		
**Pulses are a good source of iron**					.54
	True^b^	15 (37)	22 (69)		
	False	26 (63)	10 (31)		
**Pulses are high in saturated fat**					.65
	True	8 (20)	5 (16)		
	False^b^	33 (80)	27 (84)		
**Saskatchewan is a leading producer of pulses**					.03
	True	28 (68)	30 (94)		
	False^b^	13 (32)	2 (6)		
**Pulses can be used in baking**					.17
	True^b^	37 (90)	32 (100)		
	False	4 (10)	0 (0)		
**Which picture contains beans?**					.17
	Identified correct bean image	38 (93)	32 (100)		
	Did not identify correct bean image	3 (7)	0 (0)		
**Name the pulse in the provided pictures (chickpeas)**					.06
	Answered correctly	27 (66)	28 (88)		
	Not answered correctly	14 (34)	4 (12)		
**Name the pulse in the provided pictures (lentils)**					.048
	Answered correctly	22 (54)	26 (81)		
	Not answered correctly	19 (46)	6 (19)		
**Name the pulse in the provided pictures (beans)**					.09
	Answered correctly	38 (93)	32 (100)		
	Not answered correctly	3 (7)	0 (0)		
**Name the pulse in the provided pictures (peas)**					.47
	Answered correctly	34 (83)	24 (75)		
	Not answered correctly	7 (17)	8 (25)		

^a^Mann-Whitney *U* test; *P*<.05 was considered statistically significant.

^b^Correct answers.

### Attitudes Toward Pulse Consumption and Its Benefits

Information about participant attitudes is presented in Table 4. A higher score (maximum 9) indicates a greater level of positivity toward pulse consumption. The median attitude scores at baseline and study end for all participants were 6 and 7, respectively (*P*=.95). Even though there was not a statistically significant difference between baseline and study end scores, except for question 1, which showed a significant difference, the percentage of students who had a positive attitude toward pulse consumption increased for 7 of 9 attitude questions at the end of the intervention (ie, questions 1, 2, 3, 4, 5, 8, and 9; Table 4).

**Table 4:** Attitude score of students toward pulse consumption at baseline and study end.

Attitude question			Baseline (n=41), n (%)		Study end (n=32), n (%)		*P* value^a^
**1. “Pulses are healthy food for growth and development of your body””**							.05
	Disagree^b^	9 (22)		2 (6)			
	Agree^c^	32 (78)		30 (94)			
**2. “You would eat pulses if your parents serve them”**							.28
	Disagree	11 (27)		4 (12)			
	Agree	30 (73)		28 (88)			
**3. “You would eat pulses if your parents eat them too”**							.28
	Disagree	15 (37)		7 (22)			
	Agree	26 (63)		25 (78)			
**4. “Pulse-based dishes are tasty food”**							.99
	Disagree	19 (46)		12 (38)			
	Agree	22 (54)		20 (62)			
**5. “Eating pulse-based food would give you more energy to work”**							.83
	Disagree	20 (49)		11 (34)			
	Agree	21 (51)		21 (66)			
**6. “You would eat pulse if your parents encourage you”**							.50
	Disagree	12 (29)		11 (34)			
	Agree	29 (71)		21 (66)			
**7. “You would eat pulse if your teachers encourage you”**							.37
	Disagree	17 (41)		19 (59)			
	Agree	24 (58)		13 (41)			
**8. “You would like to taste new pulse-based dishes that you haven’t tried before”**							.63
	Disagree	13 (32)		10 (31)			
	Agree	28 (68)		22 (69)			
**9. “You would eat pulses if they had a more attractive appearance”**							.49
	Disagree	21 (51)		11 (34)			
	Agree	20 (49)		21 (66)			

^a^Mann-Whitney *U* test; *P*<.05 was considered significant.

^b^“Strongly disagree,” “disagree,” and “not sure” were grouped as “disagree.”

^c^“Agree” and “strongly agree” were grouped as “agree.”

### Practices Regarding Pulse Consumption by Students

Overall, 81% (26/32) of students at baseline and 97% (31/32) of students at study end reported they ate at least one type of pulse. Table 5 shows the type of pulses most often consumed by participating students at baseline and study end. Beans were the most-consumed pulses (59% of students at baseline and 62% of students at study end; *P*<.001).

Consumption of various pulse-based dishes at baseline and study end are also reported in Table 5. Consumption of various pulse-based foods improved at study end for most of the food items among high school students compared to baseline. For 7 of the 9 pulse-based dishes, there were fewer students who reported not consuming them at study end compared to baseline. In addition, for 6 of the 9 pulse-based dishes, there were improvements in the number of students who consumed them 1 time a week or more.

Students were also asked about the types of pulse-based food products that they would like to have available in their school cafeteria. In total, 16% (5/32) and 34% (11/32) of students wanted to have pulse-based bread or baked products available in their school cafeteria at baseline and study end, respectively. Furthermore, 22% (7/32) and 34% (11/32) of students wanted to have pulse-based soup available in their school cafeteria at baseline and study end, respectively.

**Table 5:** Types of pulses consumed by students, students who never consumed each pulse-based dish in the past month, and students who consumed each pulse-based dish 1 time per week or more during the past month at baseline and study end (n=32).

		Baseline, n (%)	Study end, n (%)
**Types of pulses consumed by students**			
	Split peas	3 (9)	3 (9)
	Beans	19 (59)	20 (62)
	Chickpeas	4 (12)	8 (25)
	Lentils	4 (12)	7 (22)
**Pulse-based dishes never consumed in past month**			
	Baked beans	14 (44)	14 (44)
	Soup	10 (31)	4 (12)
	Chili	7 (22)	8 (25)
	Dips/spreads	19 (59)	11 (34)
	Mixed dish	7 (22)	7 (22)
	Bread/baked products	15 (47)	13 (41)
	Salad	15 (47)	13 (41)
	Side dish	12 (38)	7 (22)
	Beverage	26 (81)	20 (62)
	Pulse-rich protein bar	16 (50)	12 (38)
**Pulse-based dishes consumed 1 time per week or more in past month**			
	Baked beans	1 (3)	7 (22)
	Soup	8 (25)	12 (38)
	Chili	7 (25)	6 (19)
	Dips/spreads	6 (19)	7 (22)
	Mixed dish	13 (41)	14 (44)
	Bread/baked products	11 (34)	10 (31)
	Salad	4 (12)	8 (25)
	Side dish	10 (31)	9 (28)
	Beverage	1 (3)	1 (3)
	Pulse-rich protein bar	6 (19)	7 (22)

### Barriers to Consumption of Pulse-Based Foods

At baseline, the median total barrier score was 0 and at study end, it was 1. There was no significant difference between baseline and study end scores (*P*=.63). Parents not cooking or consuming pulses at home, not liking the taste of pulses, and often preferring food other than pulses were the most commonly reported barriers (Table 6).

**Table 6:** Barriers reported by students regarding pulse consumption at baseline and study end.

Item			Baseline (n=41), n (%)		Study end (n=32), n (%)		*P* value^a^	
“**Pulse-based dishes upset my stomach, so I choose not to eat them”**								.40
	Disagree^b^	38 (93)		31 (97)				
	Agree^c^	3 (7)		1 (3)				
“**I do not like the taste of pulse-based dishes”**								.97
	Disagree	38 (93)		28 (88)				
	Agree	3 (7)		4 (12)				
“**I do not eat pulse-based dishes because they take too much time to eat”**								.33
	Disagree	41 (100)		32 (100)				
	Agree	0 (0)		0 (0)				
“**I do not eat pulse-based dishes because I want to eat something else”**								.68
	Disagree	31 (76)		25 (78)				
	Agree	10 (24)		7 (22)				
“**I am still hungry even after having pulse-based dishes, so I chose not to eat pulses”**								.40
	Disagree	38 (93)		30 (94)				
	Agree	3 (7)		2 (6)				
“**I do not eat pulses because my parents do not eat them”**								.76
	Disagree	35 (85)		30 (94)				
	Agree	6 (15)		2 (6)				
“**I do not eat pulses because my parents do not cook them”**								.49
	Disagree	29 (71)		24 (75)				
	Agree	12 (29)		8 (25)				
“**I do not eat pulse-based dishes because it makes my hands greasy”**								.46
	Disagree	41 (100)		30 (94)				
	Agree	0 (0)		2 (6)				

^a^Mann-Whitney *U* test; *P*<.05 was considered significant.

^b^“Strongly disagree,” “disagree,” and “not sure” were grouped as “disagree.”

^c^“Agree” and “strongly agree” were grouped as “agree.”

### Practices Regarding Pulse Consumption by Students

Five themes emerged regarding the experience of educators on the implementation of the PMPS intervention for high school students. The themes were (1) teacher experience of teaching PMPS, (2) the feasibility of integrating PMPS into the high school curriculum, (3) lessons or activities that need to be added, (4) changes in student eating behavior, and (5) use of PMPS resources and challenges in delivering PMPS in the future.

### Teacher Experience of Teaching PMPS

Both teachers and the practicum student had a good experience and impression regarding teaching the PMPS. They thought that the resources were informative and local. One of the teachers described it as follows: “the information there, it’s condensed, it’s accurate, it’s Saskatchewan based, it’s local...” Further, the teacher elaborated that the experience of using pulses for cooking was “cheaper” and a “comparative to animal source of protein” and described it as “budget friendly.”

### Feasibility of Integrating PMPS Into the High School Curriculum

The teachers believed that the content of this intervention can be integrated into the high school curriculum. One of the teachers said, “I could actually blend it with my grade 10.... It works very well in the curriculum that exists in Saskatchewan right now.” The other teacher said that the “program aligns with the new Canada’s Food Guide...there is definitely a place for it now.” In addition, the teachers suggested posting the material on the division or Saskatchewan curriculum websites to share it with others.

### Lesson or Activities That Need to Be Added

One of the teachers suggested incorporating activities on cost of ingredients, nutrient comparison of pulses versus animal-sourced foods, additional pulse-cooking opportunities, and debate and discussion about the benefits of pulses among students in future lessons. In addition, one of the teachers suggested having 1 more lesson on food security in relation to food waste.

### Change in Student Eating Behavior

The teachers believed that students recognized the nutritional and environmental benefits of pulses. The teachers observed that most students liked the taste of pulses. However, they felt that some students might not eat pulses at home due to their parents’ lack of information about the health benefits of pulses. Further, one of the teachers suggested including pulses in the school cafeteria so that more students would eat them.

### Use of PMPS Resources and Challenges in Delivering PMPS in the Future

The teachers had an interest in using the PMPS resources in their courses in the upcoming semesters. They liked the materials and thought they were “effective” and “easy to use.” A challenge mentioned by one of the teachers regarding using the PMPS material in the future was “how to get kids to engage in the material,” as she thought the materials were insufficiently interactive.

## Discussion

### Principal Findings

This study aimed to pilot-test a school-based intervention with a focus on pulses designed using a food literacy framework. This pilot intervention contributes evidence to help increase the consumption of pulses among high school students. This information is important, as the consumption of pulses among young adults (aged 18-34 years) is lower than other age groups in Canada [7,8].

Our findings show the possibilities of integrating pulse education in the high school curriculum. Nearly all students could identify and name beans, and more than 75% of the students could identify and name chickpeas, lentils, and peas at study end. In addition, most students knew the health and nutritional benefits of pulses at study end. This pilot study found that even a short intervention could promote immediate increases in pulse-related knowledge. Our data show that the students had knowledge about pulses and their benefits at baseline; however, at study end, the percentage of students who answered the questions correctly increased for most questions. Improving student knowledge of healthy diets is an important first step for behavior change, as previous research has shown a positive relationship between knowledge about healthy foods and healthy food behaviors [27]. There is an added advantage to promoting changes at a younger age: healthy food choices that are developed at a young age might be more sustainable in the long term. A study in Australia on adolescents that assessed the perspectives of students regarding food literacy found that adolescents thought that food and nutrition knowledge was important to improve their dietary behaviors [3]. Our findings are also in line with a school-based study in the United States that aimed to assess the impact of a 3-week, garden-based pulse nutrition intervention incorporated in the biology curriculum for grade 4 students. This study also found significant improvement in student knowledge and preferences regarding pulses at the end of the intervention [28]. Furthermore, the frequency of consumption of some pulse-based foods showed improvement, as the number of students who reported they consumed each of the pulse-based dishes 1 time per week or more increased at the end of the intervention.

Barriers or factors that limit the consumption of pulses among adolescents were identified at both baseline and study end. Students identified more barriers to pulse consumption at study end, which could be a result of increased knowledge. The most commonly identified barrier to pulse consumption was “I do not eat pulses because my parents do not cook them”; in total, 25% of students reported this barrier at study end. This suggests strong parental influence on the food choices of adolescents [29]. Additionally, 21.9% of students at study end agreed with the statement “I do not eat pulse-based dishes because I want to eat something else.” This finding is similar to a study in the state of Washington that identified barriers to using pulses in school cafeterias [30]. The authors of that study found that a lack of student preference for pulses was a reason for not serving them in the school cafeteria [30]. However, during the teacher interviews in our study, introducing pulses in the school cafeteria was one of the suggestions given by the teachers to improve consumption of these foods among high school students.

Teachers confirmed that students were starting to realize the nutritional, economic, and environmental benefits of consuming pulses and that pulses are a “budget-friendly” food. The teachers also suggested that adding more activities, such as debate, would strengthen the delivery of the intervention. They also wanted an increased number of interactive and reflective activities. In addition, they believed that the PMPS intervention aligns with the new Canada’s Food Guide, which creates room for integrating these lessons into the high school curriculum. Further, they indicated their interest in using the PMPS resource with few modifications in the upcoming semesters.

### Strengths and Limitations

We did not detect statistical differences in most attitude and practice variables. This finding could be due to the short duration of the intervention and the small sample size. Bai and colleagues [31] suggested repeated exposure as a strategy to improve vegetable consumption among children. This strategy could be considered for promoting pulse consumption among high school students. The main strength of this study was the collaboration between the teachers and the university researchers in developing and implementing the study. All educational materials were jointly developed by the teachers and research team using a food literacy framework. A limitation of the study was its small sample size, which lacks representativeness of high school students. Although it would have been beneficial to include schools with different sociodemographics, this study specifically aimed to focus on students with similar sociodemographics, which could be another limitation of this study. Variance in lesson delivery methods (ie, teacher vs researcher) could also be considered a limitation of our study. In addition, the students were all taking a food-related class, which suggests an interest in food. Our results may not have been the same among students who were not taking food-related classes. We observed decreased percentages of students that knew pulses are a good source of protein and correctly identified the picture of peas, which could be a result of students who dropped out of the study-end survey. Our study mainly focused on knowledge, attitudes, and practices, as well as finding possible barriers to consumption, related to pulses among high school students. Thus, our study did not assess all food literacy constructs, such as the overall capability of students to plan and manage pulse-based food products. Future studies are needed to test the feasibility of scaling up such nutrition interventions within the high school setting and to assess the long-term impact of the PMPS intervention on nutrition knowledge, attitudes, and behaviors.

### Conclusions

Despite the lack of statistically significant results for most variables, our pilot study shows promising results for pulse education tailored to high school students that is supported by a food literacy framework in improving knowledge, attitudes, and consumption of pulses. Improving knowledge and skills regarding pulse and pulse-based foods is important for adolescents to establish healthy dietary behavior that continues during adulthood. As many students indicated that “parents do not cook pulses” was a barrier, it is also important to find ways to reach parents in pulse education to further improve the use of pulses in the household. Lessons learned from this pilot intervention can be applied to other high school settings to help encourage positive changes regarding knowledge, attitudes, and practices related to healthy eating. Future research should evaluate the effectiveness of food literacy interventions in promoting pulse foods among high school students at a large scale, as well as examine the long-term impact.

## Appendix

Multimedia Appendix 1 In-depth interview.

## Abbreviations

PMPS: Pulses Make Perfect Sense
USask: University of Saskatchewan

## Data Availability

The data sets used for analysis in this study are available from the corresponding author on reasonable request.
