# Supplementary material for: Pulse-Based Nutrition Education Intervention Among High School Students to Enhance Knowledge, Attitudes, and Practices: Pilot for a Formative Survey Study
Source: JMIR Form Res. 2023 May 31;7:e45908. doi: 10.2196/45908 (PMC10267791; doi:10.2196/45908)
Supplement: Multimedia Appendix 1 [file formative_v7i1e45908_app1.docx]

**In-depth Interview**

**Respondent NO.:**

**Participant Name:**

**Site:**

**Date of interview:**

**Interviewer Name:**

1. Please describe your experience teaching the Pulse Positive Program. Did you enjoy teaching the Pulse Makes Perfect Sense (PMPS)? What did you enjoy the most? What did you enjoy the least?

2. Can you describe the students reaction to the Pulse Positive program. What did they appear to enjoy the most? What did they appear to enjoy the least?

3. And what did they appear to enjoy the least?

4. Is there any lesson or an activity that you believe need to be added, removed or improved? Please explain.

5. What are your thoughts on the Pulse Positive program as a tool to teach high school students about pulse and healthy diet.

6. What are your thoughts on the Pulse Positive lessons helping to change the students eating behavior.

7. Can you talk about your thoughts on the feasibility of integrating the Pulse Positive Program into the existing curriculum?

8. Do you have any suggestion to help in improving the acceptability of the Pulse Positive educational resource? By students? By teachers?

9. Do you plan to use the lesson in your teaching next year? Please explain

10. What challenges did you foresee in delivering the Pulse Positive program in the future? Any suggestion how the challenges can be overcome?

11. We are almost near to end to our interview. Is there anything else you would like to add about anything we spoke about today?

12. My questions are done. Do you have any question for me?

R01: No. I think we are good. we have asked all the way long (laugh). So, I think, I think I am

Thank you so much for your time.
